# Reusable tourniquets. An underestimated means for patient transfer of multi-resistant bacteria

**DOI:** 10.1186/1753-6561-5-S6-P38

**Published:** 2011-06-29

**Authors:** T Gottlieb, T Phan, EYL Cheong, G Sala, S Siarakas, A Pinto

**Affiliations:** 1 Microbiology and Infectious Diseases, Concord Hospital, Concord, Australia

## Introduction / objectives

We sought to investigate the use of reusable tourniquets as potential sources of MRO transmission.

## Methods

100 reusable tourniquets were collected over 10 weeks in a 503-bed Sydney teaching hospital. Tourniquets were incubated overnight in BHI enrichment broth and subcultured.

## Results

The colonisation rate was 78% (78/100). Ten grew non multi-resistant Gram- positives - MSSA (1) and Enterococcus species (9), 17 grew commensals. Non multi-resistant Gram-negatives grew in 38 specimens: Pseudomonas species (13) and ‘coliforms’ (26). MROs were found on 25% of tourniquets, including 3 from MRO isolation rooms. An IMP-4 positive *E. cloacae* and an ESBL *E. cloacae* were isolated from a single tourniquet each. MRSA was isolated from 14; vanB *E. faecium* was isolated from 18 and vanA *E. faecalis* from a single tourniquet. MRSA and VRE were isolated together from nine tourniquets, and 24 tourniquets grew either one. Van B positive *E.faecium* were typed using DiversiLab rep-PCR system This revealed five clusters without adominant clone. Six of 9 tourniquets from ICU grew at least one MRO. MROs were isolated throughout the 10 week period from a wide variety of locations including general wards, ICU, Burns, theatre anaesthetic bay and the blood collection unit.

## Conclusion

Reusable tourniquets are frequently colonised with MROs and may be a potential source of cross-transmission. Using broth enrichment, 24% harboured either MRSA or VRE. Astourniquets are carried from ward to ward by hospital staff and used repeatedly, they may become a ‘sleeper’ mechanism for unrecognised hospital MRO transmission. They are also a surrogate marker for environmental colonisation and deficiencies in hospital cleaning. Continued use of reusable tourniquets may not be justified in the current hospital setting.

## Disclosure of interest

None declared.

